# Recovery of Li(Ni_0.33_Mn_0.33_Co_0.33_)O_2_ from Lithium-Ion Battery Cathodes: Aspects of Degradation

**DOI:** 10.3390/nano9020246

**Published:** 2019-02-12

**Authors:** Tim Sieber, Jana Ducke, Anja Rietig, Thomas Langner, Jörg Acker

**Affiliations:** Department of Physical Chemistry, Brandenburg Technical University Cottbus-Senftenberg, Universitätsplatz 1, D-01968 Senftenberg, Germany; tim.sieber@b-tu.de (T.S.); jana.ducke@b-tu.de (J.D.); anja.rietig@b-tu.de (A.R.); thomas.langner@b-tu.de (T.L.)

**Keywords:** lithium-ion, nickel–manganese–cobalt oxide (NMC), leaching, recycling, recover, degradation, SEM-EDX, Raman spectroscopy

## Abstract

Nickel–manganese–cobalt oxides, with LiNi_0.33_Mn_0.33_Co_0.33_O_2_ (NMC) as the most prominent compound, are state-of-the-art cathode materials for lithium-ion batteries in electric vehicles. The growing market for electro mobility has led to a growing global demand for Li, Co, Ni, and Mn, making spent lithium-ion batteries a valuable secondary resource. Going forward, energy- and resource-inefficient pyrometallurgical and hydrometallurgical recycling strategies must be avoided. We presented an approach to recover NMC particles from spent lithium-ion battery cathodes while preserving their chemical and morphological properties, with a minimal use of chemicals. The key task was the separation of the cathode coating layer consisting of NMC, an organic binder, and carbon black, from the Al substrate foil. This can be performed in water under strong agitation to support the slow detachment process. However, the contact of the NMC cathode with water leads to a release of Li^+^ ions and a fast increase in the pH. Unwanted side reactions may occur as the Al substrate foil starts to dissolve and Al(OH)_3_ precipitates on the NMC. These side reactions are avoided using pH-adjusted solutions with sufficiently high buffer capacities to separate the coating layer from the Al substrate, without precipitations and without degradation of the NMC particles.

## 1. Introduction

The expanding market for electric vehicles requires lithium-ion batteries (Li-ion batteries), as these are energy storage devices with high power, high capacity, high charging rates, and long life stability. The most common cathode materials for Li-ion batteries in electric vehicles [1] belong to a group of layered mixed transition metal oxide compounds with rhombohedra symmetry (D_3d_^5^ space group), LiNi*_x_*Mn*_y_*Co_1−*x*−*y*_O_2_ (with (*x*+*y*) ∈ [0,1]) [2]. Among them, the most popular and widely used material in Li-ion batteries is the phase LiNi_0.33_Mn_0.33_Co_0.33_O_2_ (NMC). 

Global demand for powerful, rechargeable Li-ion batteries, particularly for electric vehicles, has increased the demand for essential elements Co, Ni, Mn, and Li. For years, Co has been the most valuable and critical raw element needed in battery metals [3]. In 2005, 25% of the end products made with cobalt in the EU were used in the manufacturing of battery chemicals, and this value had increased to 42% in 2015 [4]. Therefore, end-of-life Li-ion batteries have become an essential secondary source to cover the Co requirements and for other necessary elements. 

There are two basic technologies for the recycling of spent Li-ion batteries: The pyrometallurgical and the hydrometallurgical process [5,6,7,8,9,10,11,12,13,14]. The pyrometallurgical route is a smelting process in which spent Li-ion batteries are entirely melted down without further pretreatment. Organic materials are needed as fuel to maintain the melting process. A molten metal phase consisting of Co, Cu, and Ni is obtained from the smelter and leached in acid solution after cooling. After separation and chemical precipitation, the metals are recovered as inorganic salts. Al, the substrate material of the cathode, Li, and Mn are oxidized, and they are combined with other metal oxides as a slag that can be treated to recover Li. Recently, a combined route consisting of mechanical treatment (comminution of the cathodes and mechanical separation of the Al substrate foils and NMC) and hydrometallurgical treatment (dissolution of the NMC and metal recovery therefrom) was described [14]. The pyrometallurgical route is energy-intense due to the high temperatures required. It emits dust and hazardous gaseous compounds and results in a significant loss of materials. The hydrometallurgical route comprises the leaching of dismounted or shredded Li-ion batteries in strong inorganic acids to dissolve any metals and battery materials. The major challenge in this process is the separation of the ions from the concentrated metal ion liquor, mainly by selective precipitation, electrochemical deposition, or other techniques, such as solvent extraction or ion exchange. As in the pyrometallurgical route, the metals are obtained as inorganic salts. Losses typically occur from insufficient leaching and the precipitation/separation efficiencies of the metal salts. 

**Figure 1:**
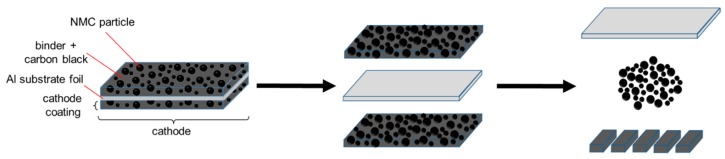
Scheme for the functional recycling of cathodes from Li-ion batteries to recover LiNi_0.33_Mn_0.33_Co_0.33_O_2_ (NMC) for re-use.

Our motivation was to avoid the pyrometallurgical and hydrometallurgical recycling routes, as well as their specific drawbacks. In this paper, we described the basics of an alternative approach to recover NMC while preserving its chemical, physical, and morphological properties, with a minimal use of chemicals. This approach, which is designated as functional recycling (Figure 1), can be applied to the cathodes of dismantled and separated end-of-life Li-ion batteries, as well as to residues or scrap from the production of cathodes. Based on the known design of a cathode in Li-ion batteries (Figure 1), the first step in functional recycling is the complete removal of the cathode coating (which consists of NMC, a binder, and conductive carbon black) from the Al substrate. This requires arranging for the medium to be as minimally reactive as possible toward the NMC or the Al substrate foil, and to keep the contact time with the medium as short as possible, so that the NMC particles do not experience any degradation. The development of such a procedure was presented in this work. Basically, this treatment is followed by a second step, not considered here, in which the mechanically separated coating is dried and mechanically comminuted to release and separate the NMC particles from the binder/carbon black mixture. 

## 2. Materials and Methods 

NMC material and cathodes: The chemical composition of the NMC material used in this study was determined by an inductively coupled plasma-optical emission spectrometer (ICP-OES) to be Li_0.945±0.007_Ni_0.336±0.001_Mn_0.331±0.001_Co_0.333±0.001_O_2_, which was close to the ideal NMC stoichiometry. The NMC consisted of spherical agglomerates (c.f. Section 3.1), designated as secondary particles, with diameters between 5 µm and 15 µm, which were constituted from primary particles with diameters of 0.5–1 µm (c.f. Section 3.1). The studied cathodes consisted of 20 µm thick Al foils, and they were coated with a layer consisting of NMC, the organic binder polyvinylidene fluoride (PVDF), and carbon black as a conductive additive. Two different cathodes were studied with a mean single-sided coating layer thickness of 50 µm and 22 µm, and they were designated as cathode 1 and cathode 2, respectively. For cathode 1, the mass fraction of Al amounted to 14.1% of the total weight, and the total Li content was 56.84 mg per 1 g cathode. The mass fraction of Al in cathode 2 amounted to 27.4%, and the total Li content was 45.64 mg per 1 g cathode. A mechanically shredded fraction, as well as a preselected fraction of the cathodes with a mass fraction of 7.0% Al, were used. 

Chemicals and reagents: All acids (HCl, HNO_3_) and chemicals (citric acid C_6_H_8_O_7_, KH_2_PO_4_, K_2_HPO_4_, NaOH, Na_2_CO_3_, and NaHCO_3_) used in this study were of analytical grade and were purchased from Merck (Darmstadt, Germany). Solutions of C_6_H_8_O_7_, KH_2_PO_4_, K_2_HPO_4_, and NaOH were prepared by dissolving the chemicals in deionized water (pH between 5.8 and 6.5, 18MΩ cm^−1^, Milli-Q, Darmstadt, Germany). The H_2_PO_4_^−^/HPO_4_^2−^ buffer solutions were prepared by mixing solutions of KH_2_PO_4_ (*c* = 66 mmol L^−1^) and K_2_HPO_4_ (*c* = 66 mmol L^−1^). Li, Ni, Mn, Co, Al, and S single-element standard solutions from Merck were used to prepare multielement calibration standards for ICP-OES measurements. The calibration standards for the measurements by ion chromatography (IC) were prepared from an SO_4_^2−^ standard solution from Merck.

Leaching experiments: The cathode foils were gently cut with scissors into square pieces with a size of 5 × 5 mm^2^. Pieces with a damaged coating layer or uneven edges were rejected. Weighed samples of the NMC (the cathode pieces or the shredded fraction) were put in contact with the leaching media. If not otherwise stated, the experiments were performed at 25 °C with a 50 mL volume of the leaching medium. All the leaching experiments were stirred at 400 min^−1^ using a magnetic stirrer (H+P Labortechnik AG, Oberschleißheim, Germany). During leaching, suspension samples were collected, and after filtration, the metal concentrations were analyzed by ICP-OES.

Sample digestion: The NMC starting material was dissolved in a mixture of HNO_3_, 69% (m/m), and HCl, 37% (m/m), using a high-pressure microwave digestion system (MLS Ethos Start, Leutkirch, Germany, 100 mL polytetrafluoroethylene (PTFE) vessels, maximum temperature 220 °C), and they were subsequently analyzed by ICP-OES.

Chemical analysis: An inductively coupled plasma-optical emission spectrometer (ICP-OES) with a dual-view option (iCap 6500 DUO, Thermo Scientific, Dreieich, Germany) was used to determine the elemental composition of the NMC and the concentrations of the dissolved elements. The sample introduction system was equipped with a parallel path nebulizer made of polyether ether ketone (PEEK) (MiraMist, Burgener Inc., Mississauga, ON, Canada), a cyclonic spray chamber (Glass Expansion, Port Melbourne, Victoria, Australia), and a ceramic injection tube (Glass Expansion, Port Melbourne, Victoria, Australia).

Ion analysis: Concentrations of sulfate ions were determined using ion chromatography (881 compact IC pro, Deutsche METROHM GmbH & Co. KG, Filderstadt, Germany). An METROSEP ASupp5 (Deutsche METROHM GmbH & Co. KG, Filderstadt, Germany; length 250 mm, diameter 4.0 mm) was used as a separation column, with a carbonate eluent consisting of 3.2 mmol L^−1^ Na_2_CO_3_ and 1.0 mmol L^−1^ NaHCO_3_. Samples and standard solutions were purified from metal ions using an SPE-H^+^-cartridge (MACHEREY-NAGEL GmbH & Co. KG, Düren, Germany).

Raman confocal microscopy: NMC particles were studied with a confocal Raman microscope (DXR SmartRaman, Thermo Fisher Scientific, Dreieich, Germany) in the backscattering configuration. The microscope was equipped with a 532 nm excitation laser and a 900 grooves/mm grating to record the Raman spectra in the wavenumber range of 150–1250 cm^−1^. The incident laser light, generated with a laser power of 0.5 mW, was focused on the sample surface through a 100× microscope objective. The laser spot had a diameter of 1.6 µm.

SEM-EDX measurements: The materials were studied with SEM-EDX (scanning electron microscopy energy-dispersive X-ray spectroscopy, using a ZEISS EVO M 15 (Carl Zeiss Microscopy GmbH, Jena, Germany) equipped with EDAX TEAM™ EDS (AMETEK, Weiterstadt, Germany).

## 3. Results

### 3.1. Cathode Foil in Contact with Water

**Figure 2:**
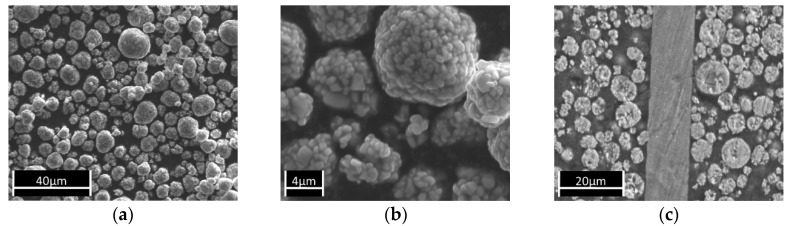
SEM micrographs of the NMC starting material: (**a**) Magnification 1000; (**b**) Magnification 2500; (**c**) Cross section of cathode foil 1.

The NMC particles in this study had a typical size between 5 µm and 15 µm (Figure 2a,b) and were processed into coatings with thicknesses typically between 22 µm and 50 µm. Figure 2c shows a SEM micrograph of a cross-section of the studied cathodes. The thickness of the Al substrate, the thickness of the coating, and the NMC content in the coating layer may vary depending on the production process. 

If a cathode is brought into contact with water, the foil may remain unchanged for days. However, if the cathode foil was mechanically comminuted or cut into square pieces with defined areas between 10 mm^2^ and 30 mm^2^, and if the aqueous suspension was strongly stirred at speeds between 400 min^−1^ and 1000 min^−1^, the coating layer completely separated from the Al carrier foil within 5 to 20 h. The time to a complete separation depended on the stirrer speed and the temperature, but it also depended, particularly, on the size of the cathode pieces, on the thickness of the coating, and on the quality of the adhesion between the coating and the Al substrate. Local damage to the coating, local lift-offs, or rough cutting edges (caused by mechanical comminution processes) strongly supported the detachment process. If the stirring was interrupted during the treatment, a weak development of gas bubbles could be observed (for an explanation, see Section 3.4), which evolved mainly from the cutting edges and from the cracks in the coating. 

### 3.2. The Release of Li and the Increase of the pH

When a cathode comes into contact with water, the pH value rises sharply in the first few minutes. The pH value of the deionized water of 5.8 to 6.5 rose to values between 9 and 11 in the course of 30–60 min. The rate and extent of the pH increase depended on the ratio of the mass of the cathode to the volume of water, on the size of the cathode pieces, and the degree of damage to the coating and the Al substrate as a result of the mechanical comminution.

**Figure 3:**
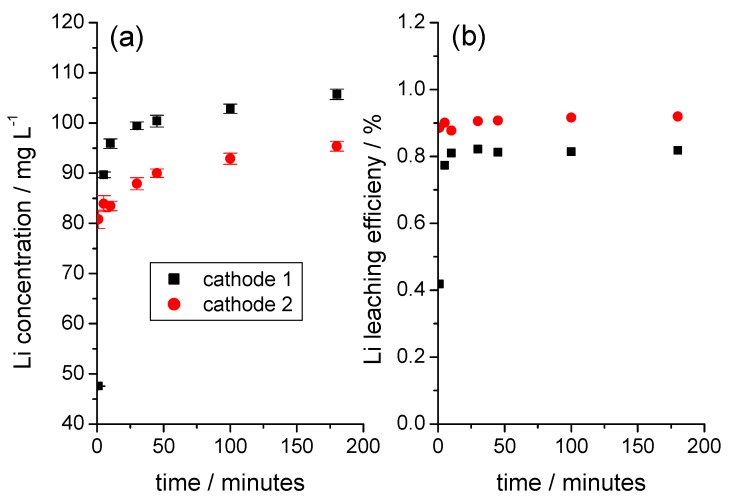
Initial period of the release of Li from two different cathode foils during immersion in water. (**a**) Solution concentration of Li as a function of time, (**b**) time dependence of the Li leaching efficiency calculated with respect to the total Li content in the sample (weight of cathode 1 sample: 1.1368 g; weight of cathode 2 sample: 1.1355 g).

**Figure 4:**
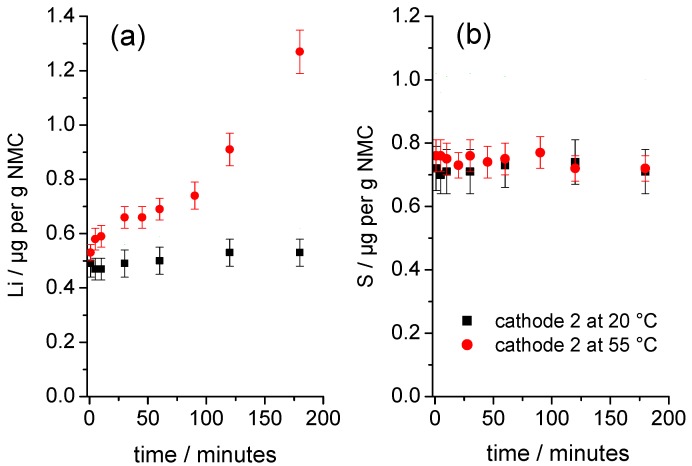
Time dependency of the release of (**a**) Li and (**b**) S from samples of cathode 2 immersed in water at 20 °C and 55 °C.

Li was released from the cathode without a time delay as soon as the cathode came into contact with water. At room temperature, the amount of Li did not subsequently increase significantly over time (Figure 3a). The amount of Li released was small in relation to the total Li content in the coating and, as in the example shown in Figure 3b, it reached a Li leaching efficiency of almost 1%. The correlation between the amount and the rate of Li release and the NMC content in the coating, as shown in Figure 4a, was only given if cathode foil pieces of a defined size, with smooth-cut edges, and without visible damage to the coating were used. 

A plausible model for the initial deintercalation of Li from NMC was recently discussed by Billy et al. [15]. A protonation of the surface of the oxidic NMC surface leads to the formation of –OH and –OH_2_^+^ species. Such a protolytic reaction at the solid–liquid interface is equivalent to an accumulation of a positive surface charge. This charge difference can be balanced by a partial internal oxidation of the transition metals and by the deintercalation of Li ions that are transferred into the aqueous solution. If this mechanism is valid for the initial contact between the NMC and an aqueous solution, then the initial increase in the pH is not caused by a generation of OH^−^ ions, but rather by a significantly reduced concentration of H^+^ ions. Since the amount of H^+^ ions attached to the NMC surface is finally limited, the amount of OH^−^ ions is limited too. 

### 3.3. The Release of Sulfur

Figure 4 shows the time-dependent development of the solution concentrations for the element’s Li and S, released from a sample of square pieces of cathode 2 in contact with water at 20 °C and 55 °C. To compare both experiments, the solution concentrations were normalized to the respective masses of the NMC in each studied cathode sample. Figure 4a shows that the initial normalized concentrations of Li at both temperatures are nearly identical. Over time, the normalized Li concentration increased only slightly at 20 °C; by contrast, the normalized Li concentration increased significantly at 55 °C, indicating that the NMC degraded significantly more at the elevated temperature.

The S concentrations (normalized to the mass of NMC in the samples) rose abruptly upon direct contact of the cathodes with water (Figure 4b) and reached similar normalized initial concentrations that remained almost unchanged over time. Surprisingly, the initial degradation of the NMC at 55 °C (Figure 4a) does not cause a further increase in the S concentration. The release of Li and S upon direct contact with water indicated that a slightly soluble Li compound was present on the surface of the NMC particles. Analysis of the solutions using ion chromatography showed that the S measured by ICP-OES was quantitatively present as sulfate ions. The molar ratio of released Li and S as sulfate *n*(Li^+^):*n*(SO_4_^2−^) for the starting material at the first measuring point was 3.14:1, which suggested that the readily soluble Li compound was mainly lithium sulfate, Li_2_SO_4_, which went directly into solution upon contact with water and did not increase the pH value. Therefore, it was assumed that the release of Li beyond the stoichiometry of Li_2_SO_4_ was essentially caused by a degradation of the NMC.

The ion chromatographic analysis of the starting material also revealed, in addition to sulfate, very low levels of phosphate and acetate near the detection limit. It is known that NMC may also contain lithium oxide and lithium peroxides [15,16,17]. However, it was assumed that their potential contribution to the release of Li was very small, and thus they were neglected. Therefore, the increase in the pH must be attributed to the degradation of the NMC. 

### 3.4. Dissolution and Precipitation of Al

**Figure 5:**
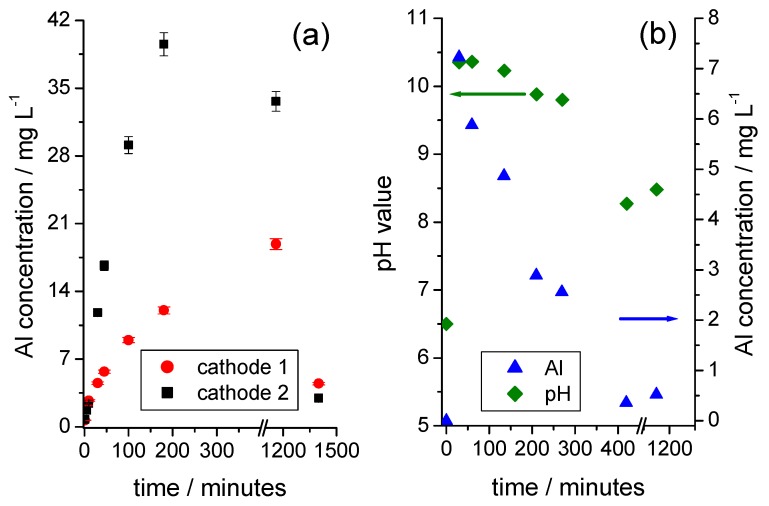
(**a**) Time-dependent development of the solution concentration of Al when the cathode foils are placed in water (sample weight of foil 1: 1.1368 g; sample weight of foil 2: 1.1355 g); (**b**) Time-dependent development of the solution concentration of Al and the pH value for a sample of a mechanically shredded cathode fraction in contact with water (sample weight: 3.0053 g).

With an increasing pH, the Al of the substrate (Figure 5a) began to dissolve, resulting in local bubble formation, preferably in places with damaged coatings and at the edges of the Al foil. According to Equation (1), from a pH value above 6, the easily soluble tetrahydroxoaluminate complex [Al(OH)_4_(H_2_O)_2_]^−^ is formed, as in Reference [18]:

(1) $$2\mathrm{Al}\;+\;2{\mathrm{OH}}^{-}+\;10H_{2}O\;\overset{}{\to }2{\left[\mathrm{Al}{\left(\mathrm{OH}\right)}_{4}{\left(H_{2}O\right)}_{2}\right]}^{-}+3H_{2}$$

This complex can be converted to the hexahydroxoaluminate complex [Al(OH)_6_]^3−^ (Equation (2)) with an increasing pH:

(2) $${\left[\mathrm{Al}{\left(\mathrm{OH}\right)}_{4}{\left(H_{2}O\right)}_{2}\right]}^{-}\overset{-H^{+}}{\underset{+H^{+}}{⇄}}{\left[\mathrm{Al}{\left(\mathrm{OH}\right)}_{5}\left(H_{2}O\right)\right]}^{2-}\overset{-H^{+}}{\underset{+H^{+}}{⇄}}{\left[\mathrm{Al}{\left(\mathrm{OH}\right)}_{6}\right]}^{3-}$$

As long as the solution is in an alkaline environment, the concentration of the dissolved Al increases with the reaction time (Figure 5a). At a certain point, the Al concentration drops significantly, and the formation of a white precipitate becomes visible. The starting precipitation is accompanied by a decrease in the pH value, as illustrated in Figure 5b, using the example of a low-aluminum, mechanically shredded cathode foil fraction. With the ongoing dissolution of Al according to Equation (1), the concentration of the hydroxide ions is exhausted and the hydroxoaluminate complexes are no longer stabilized, which is equivalent to a reverse of Equation (2). Finally, the tetrahydroxoaluminate complex reacts with the Al, and the dissolved Al is almost completely precipitated as aluminum hydroxide, Al(OH)_3_, according to Equation (3):

(3) $$\mathrm{Al}\;+\;{\left[\mathrm{Al}{\left(\mathrm{OH}\right)}_{4}\right]}^{\;-}{+\;2H}_{2}O\;\overset{}{\to }\;2\mathrm{Al}{\left(\mathrm{OH}\right)}_{3}{+\;H}_{2}$$

In some series of measurements, the pH values > 11 were measured, which might lead to a precipitation of lithium aluminum hydroxide, Li[Al(OH)_4_], according to Equation (4) [19].

(4) $${\mathrm{Li}}^{\;+}\;+\;{\left[\mathrm{Al}{\left(\mathrm{OH}\right)}_{4}\right]}^{\;-}\;\overset{}{\to }\;\mathrm{Li}\left[\mathrm{Al}{\left(\mathrm{OH}\right)}_{4}\right]$$

Reproducible, time-dependent curves for the solution concentrations of Li (Figure 3a) and Al (Figure 5a) were only obtained for defined samples of cathode foils. In these cases, the concentrations of Li and Al were in relation to each other, as long as the precipitation of Al(OH)_3_ had not occurred. Mechanically shredded cathodes with an undefined sample condition, as shown in Figure 4b, led to a significant decrease in the reproducibility of the test results.

In contrast to the dissolution of lithium, sulfate, and aluminum, only very low concentrations of Ni, Mn, and Co could be detected in solution.

**Figure 6:**
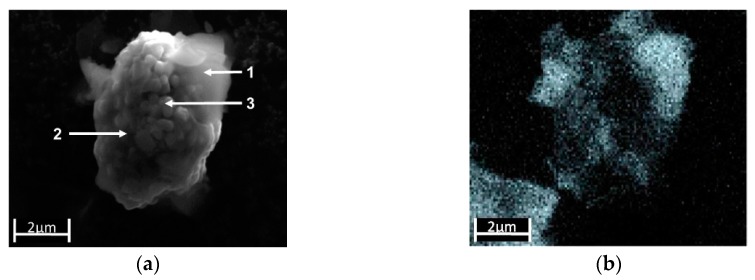

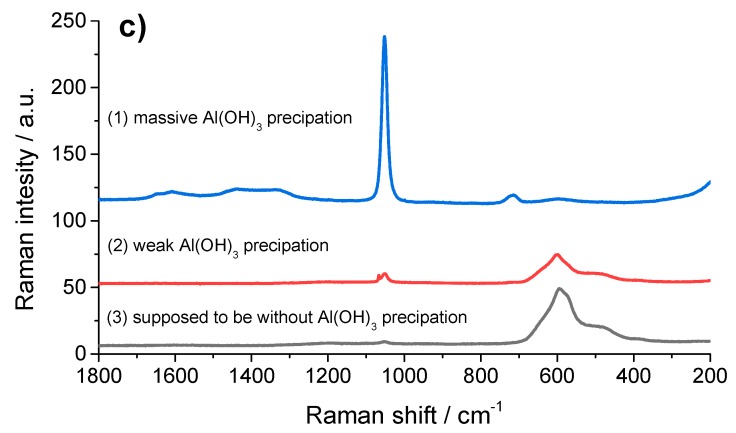
(**a**) SEM image of a NMC particle partially covered with Al(OH)_3_. (**b**) The corresponding SEM/EDX mapping of the element distributions of Al (grey: High Al intensity). (**c**) Comparison of the Raman spectra from different regions of the particle: (1) Visible precipitation of Al(OH)_3_, (2) region with apparently low Al(OH)_3_ coverage, (3) region without SEM/EDX detectable Al(OH)_3_ coverage.

Al(OH)_3_ precipitates on the NMC particles. The SEM image in Figure 6a shows a secondary particle coated with a diffuse, cloudy-like coating that covers the particles either completely (position 1) or partially (position 2). Only in the middle area (position 3) no Al(OH)_3_ coating appears to be present. The EDX element mapping for Al in Figure 6b confirmed the inhomogeneous distribution of Al across the measured particle. Compared to the high Al intensity at position 1, and a lower intensity of Al at position 2, almost no indications were found for a precipitation of Al(OH)_3_ at position 3. Figure 6c shows the Raman spectra of the three selected regions, which proved that at each position of the NMC particle, Al(OH)_3_ was detectable by the signal at 1052 cm^−1^ (the Al–O bending mode [20]), even at position 3, which was apparently assumed to be free of Al(OH)_3_ according to the EDX mapping in Figure 6b. Thus, the Al(OH)_3_ was deposited all over the entire surface in more or less thick layers on the NMC particles.

### 3.5. Use of Buffer Solutions for Separation of The Cathode Coating

The precipitation of Al(OH)_3_ on the particles can lead to a passivation, with a downgrading of the electrochemical properties that might void any re-use of the recovered NMC. One option to avoid the precipitation of Al(OH)_3_ on the NMC particles is to perform the separation of the coating from the Al substrate layer in the acidic pH range. However, this will also lead to the attack and degradation of the NMC [15]. The second option, chosen in this paper, was the separation of the coating from the Al substrate in the alkaline pH range, where a sufficiently high hydroxide ion concentration was provided to convert the dissolved Al quantitatively into the water-soluble form of a hydroxoaluminate complex. KH_2_PO_4_/K_2_HPO_4_ buffer solutions, with a high buffer capacity to maintain a constant pH value over the entire leaching period, were investigated for their potential to separate the coating from the Al substrate and to avoid a precipitation and further chemical degradation of the NMC.

To ensure a constant pH value over the treatment time, the buffer capacity (β) was estimated using Equation (5):

(5) $$\beta =\ln10\times \frac{c_{H_{2}PO_{4}^{-}}\times c_{HPO_{4}^{2-}}}{c_{H_{2}PO_{4}^{-}}+c_{HPO_{4}^{2-}}}$$

where $c_{H_{2}PO_{4}{}^{-}}$ and $c_{HPO_{4}{{}^{2}}^{-}}$ are the concentrations of dihydrogen phosphate and hydrogen phosphate present in the buffer mixture. The ratio of these concentrations was previously calculated from the Henderson–Hasselbalch equation (Equation (6)):

(6) $$\frac{c_{HPO_{4}^{2-}}}{c_{H_{2}PO_{4}^{-}}}=10^{pH-pKs}$$

for the desired pH value of the leaching solution. Under the assumption that the Li removal does not exceed a value of 5% of the Li content in the used NMC material, and in the boundary case $c_{Li^{+}}=c_{OH^{-}}$, the maximum tolerable amount of NMC per a given buffer volume can be calculated for different desired pH values—i.e., for different initial concentrations $c_{H_{2}PO_{4}{}^{-}}=c_{HPO_{4}{{}^{2}}^{-}}$.

Since the dissolution rate of the NMC increases rapidly with an increasing acid concentration [15], only one KH_2_PO_4_/K_2_HPO_4_ buffer solution in the acidic range (pH of 6) was investigated. In parallel, a test was carried out under the same conditions with deionized water as a reference. Unlike in the buffered solutions, the pH value of the deionized water changed over the entire course of the test, as shown in Figure 5b.

**Figure 7:**
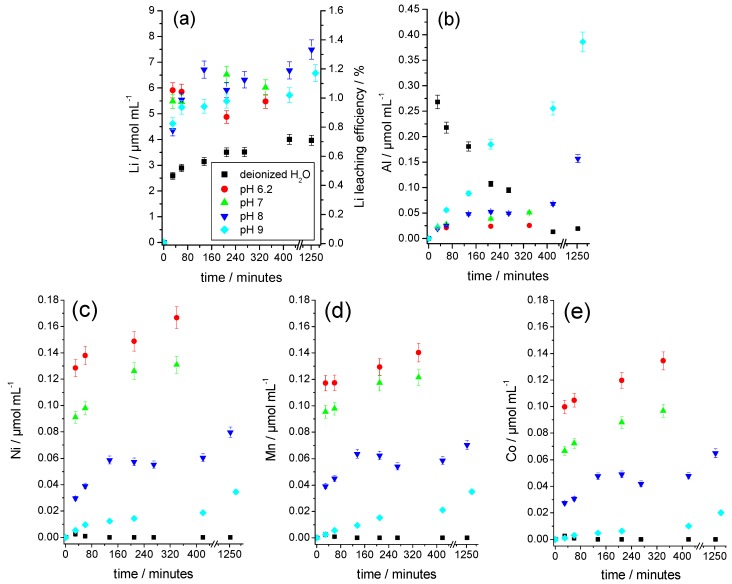
Time-dependent development of the elemental concentrations and the Li leaching efficiency in water and buffered alkaline solutions for the leaching of mechanically shredded cathodes. (**a**) Li and Li leaching efficiency, (**b**) Al, (**c**) Ni, (**d**) Mn, and (**e**) Co. Sample weights for each experiment 3.0 g.

As Figure 7a shows, Li quickly dissolves at every investigated pH value. The contents achieved after approximately 20 min were already close to the maximum concentrations after 1400 min. The Li contents in the buffer solutions were comparably high and were above the values achieved in the aqueous solution, regardless of the pH value. Figure 7b shows the behavior of Al. In an aqueous solution, the Al content initially increases sharply and then decreases again due to the precipitation of Al(OH)_3_ (which is also illustrated in Figure 5b). In the buffered solutions, however, no precipitation could be observed. In the pH range of 6 to 8, the Al concentration increased only slightly with the pH value, because the buffer solutions compensated for the initially high pH values between 9 and 11 (Figure 5b). Only at a pH value of 9 did a significant attack on the substrate occur, which coincided with a stronger evolution of bubbles. The Al concentration increased continuously and reached a higher solution concentration than in the parallel experiment with deionized water, because of the formation of the water-soluble hydroxoaluminate complexes in excess of the hydroxide ions.

Figure 7c–e shows the time dependence of the solution concentrations of Ni, Mn, and Co. These elements were only slightly dissolved in the buffered solution, whereby their solution concentrations increased continuously with a decreasing pH, as expected. A precipitation of Mn, Ni, and Co as divalent hydroxides was not plausible because their solubility constants (2.1 × 10^−13^ mol^3^·L^−3^, 5.5 × 10^−16^ mol^3^·L^−3^, and 1.1 × 10^−15^ mol^3^·L^−3^) were significantly higher than those of the much less soluble Al(OH)_3_ with 6 × 10^−33^ mol^4^·L^−4^ [21]. 

The examination of the treated NMC samples by SEM-EDX and Raman spectroscopy showed no indications for a deposition of Al(OH)_3_. Furthermore, there were no indications of degradation detected by X-ray powder diffraction, such as the formation of a phase, with a birnessite-type layered structure as observed by Billy et al. [15]. The chemical analysis of the treated samples gave stoichiometries that were identical to the initial stoichiometry within the range of uncertainty (c.f. Section 2). 

### 3.6. Secondary Particle Disintegration 

One criterion for the reuse of the recovered NMC is that the particle size and morphology should remain substantially unchanged by the treatment. So far, the removal of the coating in the buffered alkaline media did not cause any detectable change in the size or morphology of the NMC secondary particles. As shown before, the majority of the leached Li comes from the dissolution of Li_2_SO_4_, which presumably covers the outer surface of the NMC particles, and not by the Li^+^ vs. H^+^ exchange, nor by a significant degradation of the NMC particles. 

**Figure 8:**
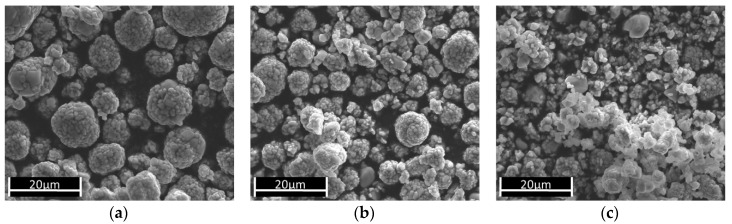
SEM images of the NMC particles (starting material after treatment with citric acid (pH 2.93, 25 °C, concentration 10^−2^ mol L^−1^) for (**a**) 30 min, (**b**) 300 min, and (**c**) 1815 min. The corresponding Li leaching efficiencies were 0.5% for a, 1.6% for b, and 2.2% for c.

However, a relationship between Li leaching efficiency and secondary particle disintegration was found. The NMC particles were treated with an aqueous citric acid solution (concentration 10^−2^ mol L^−1^, pH 2.93, 25 °C) to accelerate the dissolution process. The SEM images in Figure 8 show the change of the particles with increasing treatment duration. After 30 min and a Li leaching of 0.5%, no change was visible (Figure 8). After 300 min and a Li removal of 1.6%, the first changes in the NMC particles were visible, as the particle size was reduced, and many single primary particles or small particles (which consist of a group of a few single primary particles) increased. This evolution continued with a longer treatment time, such that after 1815 min and a Li removal of 2.2%, a large decay of the secondary particles occurred. At the same time, Ni, Mn, and Co also dissolved as a result of a chemical attack on the NMC particles. In the experiments with the buffered alkaline solutions, a maximum Li leaching of approximately 1.3% was achieved at pH 8 after 1250 min (Figure 7a). This was only slightly below the value that clearly marked the disintegration of the secondary particles during treatment with citric acid. Even if considerably longer leaching times in alkaline leaching solutions are required to achieve a Li reduction of approximately 1.6%, the experimental duration is a decisive process parameter for the recovery of the NMC. In a technical implementation, it has to be considered that the reaction conditions are far less controllable than in a laboratory experiment, and that residence times in the associated technical facilities and conveyor sections must be taken into account, which can significantly increase the contact time between the NMC and the basic medium.

### 3.7. Raman Spectroscopy Studies

Raman spectroscopy applied to the NMC-type material with a D_3d_^5^ space group can probe the Raman active M–O symmetrical stretching vibrations, the A_1g_ mode, and the O–M–O bending vibrations, the *E_g_* mode (M = Ni, Mn, Co) [2,22,23]. 

**Figure 9:**
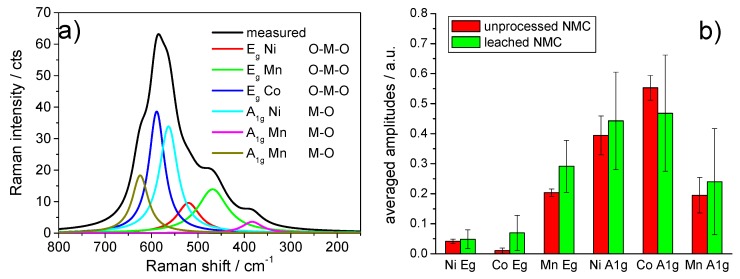
(**a**) Deconvolution of an NMC Raman spectrum and assignment of the subspectra according to Reference [2]. (**b**) Plot of the normalized subspectra amplitudes extracted from the deconvolution of Raman spectra of the NMC starting material and the NMC treated for 180 min with water. The column heights indicate the mean of the normalized areas, and the bars represent the width of scatter of the individual results.

In this study, Raman microscopy was used to detect changes to the NMC particles as a result of their treatment with water and buffered alkaline solutions. The deconvolution and assignment of the measured Raman spectra, shown exemplarily in Figure 9a, was made according to Zhang et al. [2]. Figure 9b shows a plot of the normalized peak areas for the deconvoluted modes of NMC, the particles before and after treatment with water for 180 min in the absence of Al. The Raman measurements were made at arbitrarily chosen spots on the sample of water-treated NMC. The mean peak areas for a given vibration mode were represented by the heights of the columns, and the error bars represented the spread of the individual area values. Figure 9b represents the scatter of the individual Raman spectra originating from the local inhomogeneities in the starting material and reproduced in the treated NMC material. It was obvious that a reliable statement about a possible localized degradation of the NMC particles could not be derived.

**Figure 10:**
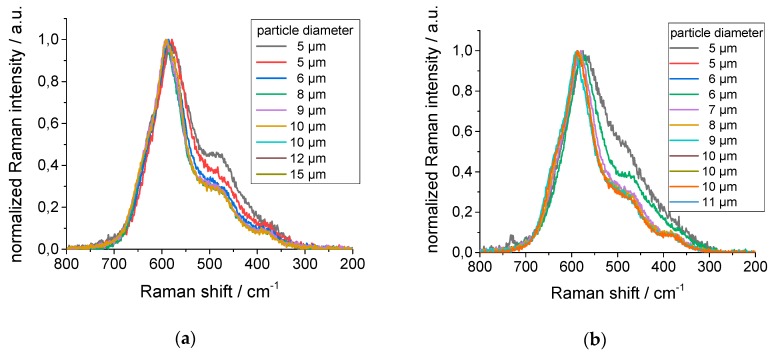
Raman spectra of the individual NMC secondary particles of different diameters (**a**) of the starting material and (**b**) after water treatment for 180 min.

The extent of local inhomogeneities underlined the normalized Raman spectra recorded from the individual NMC particles of different diameters of the starting material in Figure 10a, and after water treatment in Figure 10b. Each particle, regardless of size and even at an identical diameter, seemed to exhibit its own Raman spectrum. These studies showed the challenge in identifying suitable recovered NMC for further re-use in Li-ion batteries by Raman spectroscopy. Nevertheless, these results are the starting point for further studies.

## 4. Discussion

If NMC comes into contact with water, a protonation of the NMC surface takes place, which leads to M–OH_2_^+^ species at the surface. The enrichment of the positive charge at the surface requires a charge balance, which leads to the deintercalation of Li^+^. In practice, this refers to an exchange of Li^+^ vs. H^+^, which reduces the H^+^ ion concentration in solution and thus increases the pH value and leads to the dissolution of Al. However, not all the NMC is available for protonation due to the embedding in the PVDF polymer. Furthermore, diffusion processes, partial under-etching of the coating, and cracks and deformations can influence the protonation and deintercalation of Li, as well as the dissolution of Al. This means that the defined correlations between the solution contents of Li^+^ and Al^3+^, as well as the time-dependent leaching behavior, can hardly be reproduced to a high quality. If the Al is initially dissolved as a hydroxoalumina complex, the continued dissolution of the Al leads to a reduction in the hydroxide ion concentration, which finally leads to the precipitation of Al(OH)_3_, and possibly LiAl(OH)_4_. The insoluble Al(OH)_3_ precipitates in several or a few thick layers on the NMC particles. This passivation is considered to downgrade the electrochemical performance of the recovered NMC particles and might prevent their re-use.

The precipitation of Al(OH)_3_ on the NMC particles can be avoided if alkaline solutions with sufficient buffer capacity are used instead of water. Although an attack on the Al substrate film also occurs in the alkaline medium, the Al remains in solution in excess of the hydroxide ions, forming highly water-soluble hydroxoaluminate complexes. The alkaline medium leads to a partial attack on the NMC, which manifests itself in low solution concentrations of Ni, Mn, and Co. The compromise between the side reactions of dissolving Al and dissolving Ni, Mn, and Co is the choice of a pH value between 7 and 8. At the end of the experiments, the NMC particles are almost identical in size and morphology as the starting material used.

The amount of Li released in the experiments was small in relation to the total Li content in the studied cathode samples. Two parallel proceeding mechanisms seem to determine the release of Li: (i) The degradation of the NMC according to Billy et al. [15] and (ii) a release via the dissolution of traces of inorganic salts, in this study, presumably Li_2_SO_4_. In the alkaline buffered media, typical Li leaching efficiency values were in the range of 1%, and the maximum value obtained after the longest leaching time of 1250 min was found to be 1.3%. Although this value was low and was determined mainly by the highly soluble Li salts (Li_2_SO_4_), a higher removal of Li should be avoided. Investigations in the acid solution showed that a Li removal of approximately 1.6% disintegrates the NMC secondary particles, generating significantly smaller secondary particles, which are particles consisting of only a few primary particles, and even single primary particles. Thus, the salts present on the surface contribute significantly to the cohesion of the secondary particles. This demonstrates how sensitively the chemical treatment influences the particle size, and thus, the quality of the recovered NMC. Finally, Raman spectroscopy is typically used to characterize NMC and to evaluate the quality of the recovered NMC. However, a reasonable interpretation of the Raman spectra was limited because the NMC starting material, as well as the treated NMC, were inhomogeneous. The resulting Raman spectra of the starting material and the treated NMC exhibited such a scattered range that clear statements about the quality of the treated NMC cannot be made at the present time. Further efforts—in particular, electrochemical studies on the performance of the recovered NMC—are necessary to investigate this phenomenon.

## 5. Conclusions

This paper described the first step of an approach, designated as functional recycling, to recover NMC material from the cathodes of end-of-life Li-ion batteries or scrap from the production of cathodes, while preserving its chemical, physical, and morphological properties. The separation of the cathode coating from the Al substrate foil can be performed in a neutral or alkaline medium, with minimal mechanical treatment. However, several chemical side reactions and side effects, such as the release of Li, the formation of a passivation layer, or the disintegration of the secondary NMC particles, were identified to have a crucial effect on the quality of the recovered NMC material. Furthermore, this paper demonstrated the emerging challenges to the analytical methods and tools to detect the smallest changes in the material, to develop the most efficient and environmentally friendly route to recover NMC for re-use in Li-ion batteries.
